# *Cornukaempferia puangpeniae* sp. nov. and *C. aurantiiflora* var. *vespera* var. nov. (Zingiberaceae) from Northern Thailand

**DOI:** 10.1016/j.heliyon.2024.e41603

**Published:** 2024-12-31

**Authors:** Piyaporn Saensouk, Surapon Saensouk, Thawatphong Boonma, Chainarong Techa, Sarayut Rakarcha, Areerat Ragsasilp, Danh Duc Nguyen

**Affiliations:** a Diversity of Family Zingiberaceae and Vascular Plant for its Applications Research Unit, Department of Biology, Faculty of Science, Mahasarakham University, Kantarawichai District, Maha Sarakham, 44150, Thailand; b Diversity of Family Zingiberaceae and Vascular Plant for its Applications Research Unit, Walai Rukhavej Botanical Research Institute, Mahasarakham University, Kantarawichai District, Maha Sarakham, 44150, Thailand; c Brio Botanical Research Garden, 53 M. 5 Phikun-ok, Ban Na District, Nakhon Nayok, 26110, Thailand; d 74/1 M. 1 Dong Khu, Si Satchanalai District, Sukhothai, 64130, Thailand; e Queen Sirikit Botanic Garden, The Botanical Garden Organization, Chiang Mai, 50180, Thailand; f Diversity of Family Zingiberaceae and Vascular Plant for its Applications Research Unit, Program of Environmental and Resource Management, Faculty of Environment and Resource Studies, Mahasarakham University, Kantarawichai District, Maha Sarakham, 44150, Thailand; g Institute of Applied Technology, Thu Dau Mot University, no. 06, Tran Van on Street, Phu Hoa, Thu Dau Mot, Binh Duong, Vietnam

**Keywords:** *Cornukaempferia*, new species, Sukhothai, Taxonomy, Uttaradit, Zingiberaceae

## Abstract

Thailand hosts a diverse array of plants in the Zingiberaceae family, with over 150 endemic species, highlighting its significance in global biodiversity. The genus *Cornukaempferia* stands out for its ornamental and medicinal value. During a research expedition in Northern Thailand, a previously unknown *Cornukaempferia* species was discovered in Sukhothai province, expanding the known distribution range. It was confirmed through comprehensive taxonomic analysis as a new species, *Cornukaempferia puangpeniae*. Additionally, a new variety, *C. aurantiiflora* var. *vespera*, was identified in Uttaradit Province. The genus now encompasses eight species and two varieties with the addition of these taxa, exhibiting a notable prevalence in Thailand and highlighting its significant endemism. Particularly, *C. larsenii* is noteworthy for being native to Thailand and Laos. Detailed morphological descriptions, including diagnoses with related taxa, were provided. The species' ecological characteristics, pollen morphology, traditional uses, and conservation status were documented. A revised key to species and varieties in the genus *Cornukaempferia*, including a UPGMA cluster analysis dendrogram elucidating morphological relationships within the genus, was presented. These discoveries contribute to the botanical knowledge of the region, underscoring the importance of continued exploration and conservation efforts in preserving its rich flora.

## 1 Introduction

Thailand is renowned for its diverse array of plants within the Zingiberaceae family, encompassing 28 acknowledged genera and exceeding 400 species. These botanical treasures thrive in various habitats across Thailand, spanning terrestrial environments to epiphytic niches. Notably, over 150 of these species are endemic to Thailand [[1], [2], [3], [4], [5], [6]], underscoring the nation's pivotal role in conserving remarkable biodiversity. Among these, *Cornukaempferia* Mood & K. Larsen, initially established as a new genus from Thailand, was introduced in the Bangkok market and has gained recognition among foreign enthusiasts for its ornamental qualities. For instance, in America, it is marketed under the name “velvet butterfly,” while in Thai vernacular, it is referred to as “Proh Thong.” [[7], [8], [9], [10]]. The genus was recently revised in 2022 [10], and consists of a total of seven species, including *C. argentifolia* Boonma & Saensouk, *C. aurantiiflora* Mood & K. Larsen, *C. chayanii* Yupparach & Wongsuwan, *C. kamolwaniae* Picheans., Yupparach & Wongsuwan, *C. larsenii* P. Saensouk, *C. longipetiolata* Mood & K. Larsen, and *C. srisumoniae* P. Saensouk, Saensouk & Boonma—a recently identified new species from the easternmost part of Loei province. Significantly, six of these species are exclusively found endemic in Thailand, and their distribution covers four provinces: Uttaradit, Phitsanulok, Loei, and Phetchabun. *C. larsenii* is the only species that is naturally present in both Thailand and Laos. Moreover, there have been documented cases of these high-potential ornamental plants from this genus transitioning into horticultural use as decorative plants, with evidence of their export and trading being recorded in both traditional plant markets and online platforms. Additionally, the rhizomes of *C. aurantiiflora* and *C. longipetiolata* were recorded for medicinal purposes [1,[7], [8], [9]]. While conducting a research expedition to examine the diversity of Zingiberaceae plants in Thailand, two previously undiscovered taxa of *Cornukaempferia* were stumbled upon in Northern Thailand. This finding is significant as the area had not been previously documented within the recorded distribution range of Zingiberaceae plants. Subsequently, specimens were collected for further taxonomic investigation to classify the unidentified species precisely.

## 2 Materials and methods

**Fig. 1:**
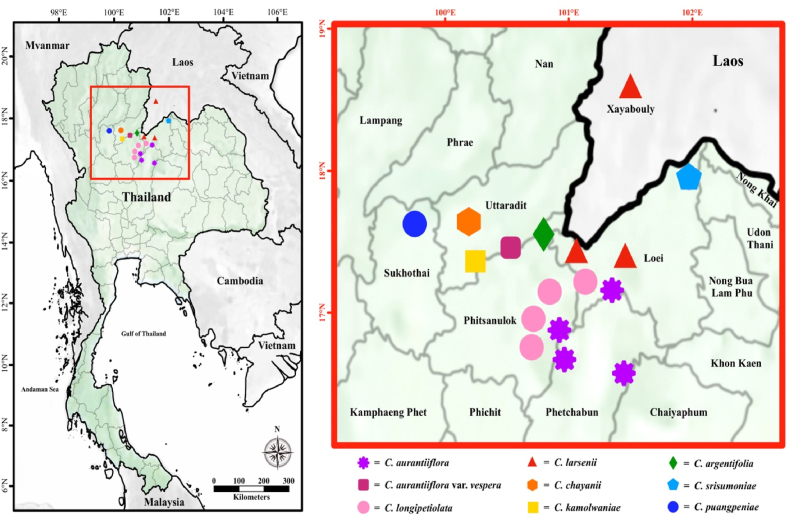
*Cornukaempferia* taxa's distribution map, with an enlarged view of the distribution area provided on the right. The distribution locations of each species are determined based on the protologue of all species within the *Cornukaempferia* genus.

**Plant materials**: Plant specimens of two taxa were gathered from Sukhothai province and Uttaradit province (Fig. 1), Northern Thailand in 2022–2023.

**Taxonomic treatment:** Meticulous measurements of morphology were carried out, utilizing 20 specimens from both preserved and living samples of each taxon, under a stereoscopic microscope (ZEISS–Stemi 2000-C, Oberkochen, Germany). The preserved specimens underwent deposition at the KKU Herbarium, the Herbarium of Faculty of Forestry (FOF), and the Mahasarakham University Herbarium. After completing the taxonomic treatment, the living specimens were planted in the garden of the Diversity of Family Zingiberaceae and Vascular Plant for Its Applications Unit, and Brio Botanical Research Garden (BBRG), while the comparative analyses were carried out with dried specimens from various herbaria (AAU!, BK!, BKF!, E!, FOF!, HNL!, KKU!, PSU!, QBG!, and US!), along with the protologues of all species in *Cornukaempferia*. The morphological characteristics of *Cornukaempferia* taxa were evaluated for similarity using the Past4 program. This involved employing the Jaccard Similarity Index and the Unweighted Pair Group Method with Arithmetic Mean (UPGMA) algorithm to generate a clustering dendrogram. Data on both quantitative and qualitative traits for each species were collected (see Table 1) and utilized in the cluster analysis to determine the species most akin to *Cornukaempferia puangpeniae*.

**Table 1:** Morphological character of *Cornukaempferia* taxa and character states with numeric codes.

No.	Characters	States
**Quantitative characters**		
1	Layer of rhizome	layers
2	Leaf-sheaths length	cm
3	Petiole length	cm
4	Leaves length	cm
5	Leaves width	cm
6	Calyx length	cm
7	Floral tube length	cm
8	Dorsal corolla lobe length	cm
9	Dorsal corolla lobe width	cm
10	Lateral corolla lobe length	cm
11	Lateral corolla lobe width	cm
12	Lateral staminodes length	cm
13	Lateral staminodes width	cm
14	Labellum length	cm
15	Labellum width	cm
16	Anther length	cm
17	Epigynous glands length	cm
**Qualitative characters**		
18	Rhizome-color of outer layer	white to yellow = 0, purple = 1
19	Rhizome-color of core	white to yellow = 0, purple = 1
20	Upper leaves color	green = 0, dark green = 1, silvery green = 2
21	Upper leaves with silver marking	no marking = 0, few = 1, numerous = 2
22	Lower surface color	green = 0, with reddish = 1
23	Bracts surface	glabrous = 0, pubescent = 1
24	Floral tube surfaces	glabrous = 0, pubescent = 1
25	Dorsal corolla lobe surfaces	glabrous = 0, pubescent = 1
26	Apex of lateral corolla lobe	rounded = 0, obtuse = 1, acute = 2
27	Staminodes surface	glabrous = 0, pubescent = 1
28	Labellum surface	glabrous = 0, pubescent = 1
29	Labellum apex	rounded = 0, acute = 1, emarginate = 2
30	Filament surface	glabrous = 0, pubescent = 1

Altitude of two new taxa from recorded tracks in the “Outdooractive” application, developed by Augmentra Ltd. in Cambridge, UK. The altitude of all existing species of *Cornukaempferia* data used to make box plots of preliminary altitude ranges is retrieved from the protologues of each taxon, our field expeditions (previously published in Saensouk et al., 2022 [10]), and data on herbarium specimen vouchers which are available at https://padme.rbge.org.uk/ZRC/data/specimens. An initial conservation status was conducted following the IUCN Red List Categories and Criteria, Version 16 [11].

**Palynology:** The pollen grains of two taxa were examined under scanning electron microscopy (SEM) and light microscopy (LM). Pollen was obtained from the alcohol-preserved material. Samples were dehydrated using an alcohol series of 70 %, 80 %, 95 %, and 100 %, respectively. For scanning electron microscopy (SEM) studies, pollen grains in absolute alcohol were air-dried and affixed to aluminium stubs using double-sided cellophane tape. Samples were then sputter-coated with a gold-palladium layer, examined, and photographed under SEM. For light microscopy (LM) studies, at least 30 pollen grains of each species were measured for various parameters, including pollen diameter (μm), length of spine (μm), and wall thickness (μm).

## 3 Results and discussion

This study introduces two newly discovered taxa from Northern Thailand, including a new species from Sukhothai Province, and a new variety from Uttaradit Province.

### 3.1 *Cornukaempferia puangpeniae* P.Saensouk, Saensouk, Boonma & Techa sp. nov

**Fig. 2:**
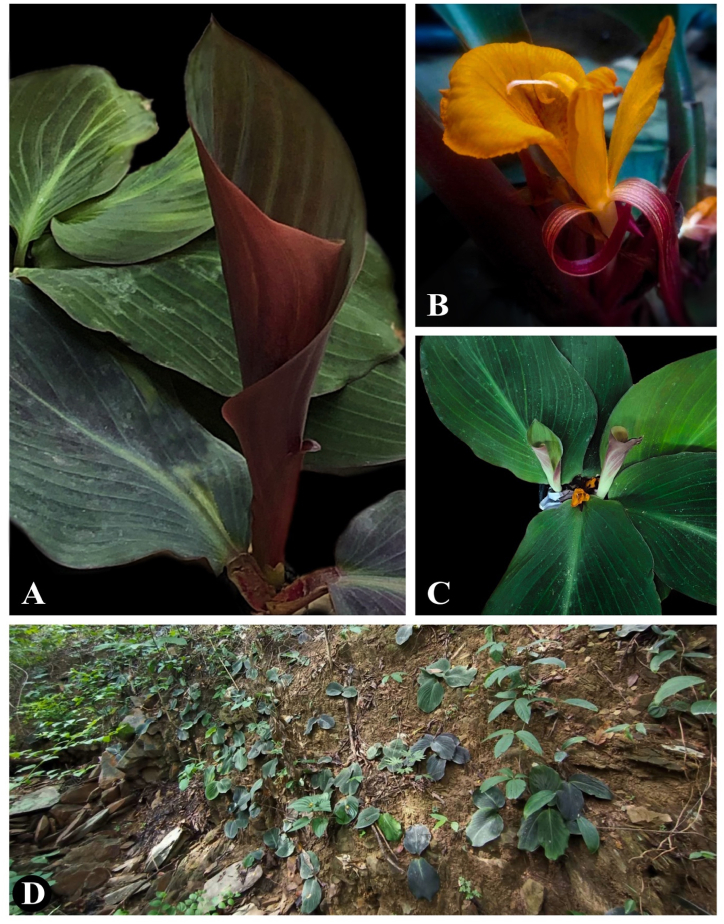
*Cornukaempferia puangpeniae* P. Saensouk, Saensouk, Boonma & Techa sp. nov.; (A) young leaf is rolled and erect before unfurling; (B) flower; (C) habit; (D) habitat (Photographs: A–B by Thawatphong Boonma, C by Surapon Saensouk, D by Chainarong Techa).

**Fig. 3:**
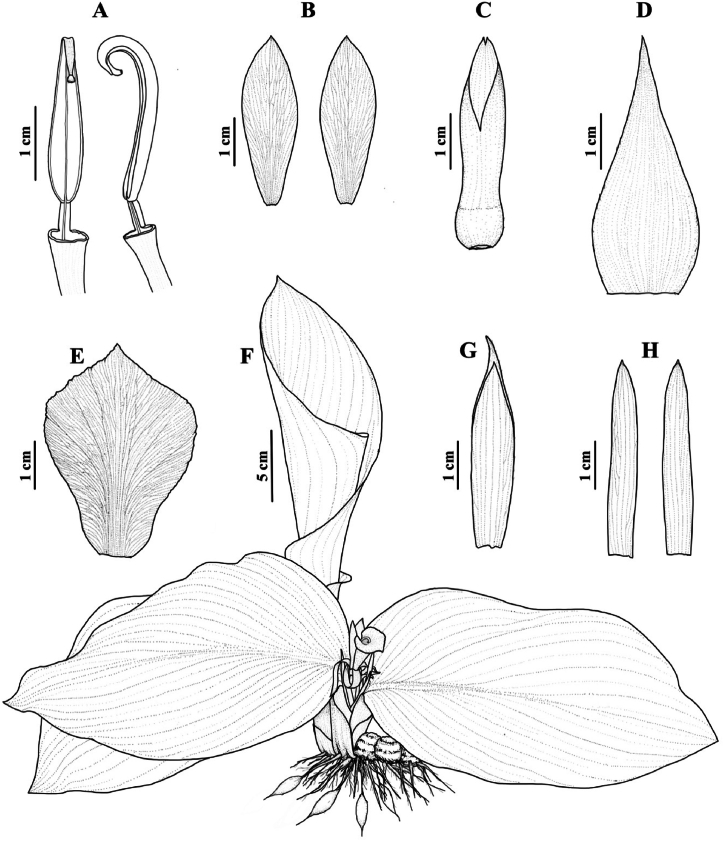
Line drawing of *Cornukaempferia puangpeniae* P. Saensouk, Saensouk, Boonma & Techa sp. nov. based on the holotype; (A) front and side view of anther with filament; (B) staminodes; (C) calyx with ovary; (D) bract; (E) labellum; (F) habit; (G) dorsal corolla lobe; (H) lateral corolla lobes (Drawn by Thawatphong Boonma).

**Fig. 4:**
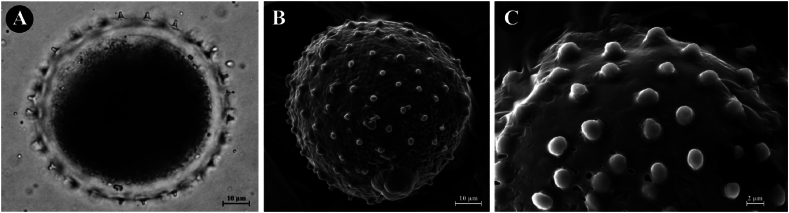
*Cornukaempferia puangpeniae* P. Saensouk, Saensouk, Boonma & Techa sp. nov.; (A) Light Microscopy (LM) of pollen grain; (B) Scanning Electron microscopy (SEM) of pollen grain; (C) Scanning Electron microscopy (SEM) of exine sculpturing.

(Table 2, Fig. 1, Fig. 2, 3(A–H), 4(A–C))

**Table 2:** Morphological comparison of *C. puangpeniae* and *C. kamolwaniae*.

Characters	*C. puangpeniae*	*C. kamolwaniae*
Tuberous root layers	2 layers, a light brown core, and an outer yellow layer	3 layers, a brownish inner core, followed by an orange-brownish middle layer, and an outer layer in yellow-oak
Leaf sheath	5–7 cm long	3.5–4 cm long
Lamina	Adaxially surface dark green with a dark reddish-purple tinge	Adaxially surface dark green without a dark reddish-purple tinge
Bracts	bracts pubescent	glabrous with a pubescent apex
Floral tube	sparsely hairy	glabrous
Staminodes	apex acute	apex rounded
Labellum	apex acute, without incision	apex rounded, with an incision 2–3 mm long
Filament	4.5–5 mm long, sparsely hairy	7–9 mm long, glabrous
Anther	2.5–2.8 cm long	1.5–1.8 cm long
Epigynous glands	c. 4 mm in length	1–2 mm in length

**Diagnosis:** Similar to *C. kamolwaniae* Picheans., Yupparach & Wongsuwan, but differs, particularly in dormancy characteristics, in having a tuberous root with two layers: a light brown core and an outer yellow layer (vs. *C. kamolwaniae*, which have a brownish inner core, followed by an orange-brownish middle layer, and finally an outer layer in yellow-oak); leaf sheath 5–7 cm long (vs. 3.5–4 cm long); lamina adaxially dark green with a dark reddish-purple tinge (vs. dark green without a dark reddish-purple tinge); bracts pubescent (vs. glabrous with a pubescent apex); floral tube sparsely hairy (vs. glabrous); staminodes apex acute (vs. apex rounded); labellum apex acute without incision (vs. apex rounded with an incision 2–3 mm long); filament 4.5–5 mm long, sparsely hairy (vs. 7–9 mm long, glabrous); anther 2.5–2.8 cm long (vs. 1.5–1.8 cm long); epigynous glands measure approximately 4 mm in length (vs. 1–2 mm in length).

**Type**: Thailand, Sukhothai Province, Si Satchanalai District, c. 220 m a.s.l., *S.Saensouk 2023-20*, August 15, 2023 (holotype KKU!, isotype FOF!).

**Description:** Perennial herb with subglobose to ovoid rhizomes, with two layers: a light brown core and an outer yellow layer, slightly aromatic, sympodial. *Roots* are fibrous, bearing ellipsoid tuberous structures, which are yellowish with a light brown core. *Bladeless sheath*, 1–3 in number, measures 2–5 cm in length and exhibits an apex that is acute to slightly mucronate, with ciliate margins. It is pubescent and displays a green coloration with a reddish tinge. *Leaf sheaths* are distichous, measuring 5–7 cm in length, and are pubescent with a reddish-brown hue. *Ligule* membranous, 2–4 mm long, emarginate. *Leaves* usually 2; *petioles* very short, 0.5–2 cm long, reddish brown; *young leaf* is rolled and erect before unfurling, then lies flat on the ground or is slightly angled; *lamina* is asymmetrical, ranging from ovate to elliptic or obovate, measuring 18–25 × 12–18 cm. It has a mucronate apex and a base that is rounded to attenuate. The margin is entire, with reddish semi-translucent hyaline, while the adaxial surface is dark green with a dark reddish-purple tinge, occasionally displaying silvery markings. The midrib is lighter green, and there are slightly embossed veins. The abaxial surface is paler green with a dark reddish-purple tinge, and both surfaces are pubescent. *Inflorescence* is terminal, bearing 3–5 flowers. The p*eduncle* is very short, measuring up to 1 cm in length. *Bract* is lanceolate, measuring 4–5 cm in length, with an acute apex, pubescent and red in color. *Calyx* is flattened tubular, 3–3.5 cm long, with a bilobed apex and an incision up to 1.5 cm long, pubescent, and reddish. *Floral tube* measures 2.6–3.2 cm in length and is sparsely hairy, pale yellowish white; *dorsal corolla lobe* is oblanceolate, measuring 4.2–4.5 × 0.8–0.95 cm, with a mucronate apex (mucro approximately 3 mm long), hooded, sparsely hairy, and red; *lateral corolla lobes* are narrowly oblong, measuring 4.0–4.2 × 0.55–0.63 cm, with slightly mucronate apices, sparsely hairy and red. *Lateral staminodes* are narrowly obovate to oblanceolate, measuring 3.2–3.5 × 0.9–1.2 cm, with acute apices, pubescent and yellowish-orange, with a pale yellow base. The *labellum* is obovate, measuring 4.3–4.6 × 3.1–3.3 cm, with an acute apex and no incision. It has a slightly fine undulate margin and is pubescent. The upper surface is deep yellowish orange with pale yellow at the base, adorned with red spots along the veins that extend about halfway to the base. The lower surface is yellowish orange with pale yellow coloring. *Stamen* 1; *filament* 4.5–5 mm long, c. 2 mm wide, pale yellow, sparsely hairy; *anther* measures 25–28 × 4.3–4.6 mm, is yellow in color and opens longitudinally, the connective extends into a long, narrow, forward-curved crest and is pubescent. *Epigynous glands* two in number, are slender, approximately 4 mm long, and yellow, with blunt apices. The o*vary* is slightly flattened and obovate, measuring 7–7.5 × 6.5–7 mm, pubescent and red in color; *style* white, glabrous; *stigma* ciliate, approximately 1.3–1.5 mm wide is yellowish and shaped like a slightly flattened inverted cone. *Fruits* and *Seeds* not seen.

**Notes**: The first shoot after dormancy typically produces a sessile petiole, while the second and third leafy shoots that emerge later tend to have slightly longer petioles, up to 2 cm in length.

**Vernacular name**: Proh Thong Puangpen.

**Etymology**: The specific epithet “*puangpeniae*” is a tribute to Prof. Dr. Puangpen Sirirugsa, a renowned botanist with expertise in Zingiberaceae. She holds the distinction of being the first Thai researcher to conduct extensive studies on the Zingiberaceae family in Thailand.

**Distribution**: This newly discovered species has been categorized as endemic to Thailand, meaning it is found exclusively inside the forests of Sukhothai Province in northern Thailand.

**Ecology**: This species can be discovered in deciduous forests that are located near watercourses. It flourishes in soil that is a mixture of sandy loam and pebbles. It thrives in a habitat with high levels of wetness and is commonly located at elevations between 200 and 300 m above sea level and is found growing alongside *Kaempferia rotunda* L. and *Globba substrigosa* King ex Baker.

**Phenology**: Flowering occurs between the periods of July and September, having almost all the anthesis happening place early in the morning (see Fig. 3).

**Palynology:** The pollen grains of *Cornukaempferia puangpeniae* are monad, inaperturate, spherical, with thick intine and thin exine with echinate sculpture, 74.12 ± 2.57 in diameter. The spine 3.25 ± 0.13 μm in length. The wall thickness 6.21 ± 0.23 μm (Fig. 4(A–C)).

**Utilization**: This new taxon has high potential for use as both an ornamental potted plant and a medicinal plant, like other species within the same genus.

**Conservation status**: *Cornukaempferia puangpeniae*, a species with a geographic range confined to less than 500 km^2^ and found in only three locations, faces significant conservation challenges. With a population of fewer than 200 mature individuals, it is classified as Endangered according to IUCN Red List Categories and Criteria, Version 16 (EN (B2aii, D1)) [11]. This status highlights the species' vulnerability to extinction due to habitat loss, degradation, and potential threats from human activities. Urgent conservation measures are needed to protect its remaining habitats, mitigate threats, and ensure the long-term survival of *Cornukaempferia puangpeniae*. Efforts such as habitat restoration, protected area management, and community engagement will be crucial in safeguarding this species and preserving its ecological role within its limited range.

### 3.2 *Cornukaempferia aurantiiflora* var. *vespera* P.Saensouk, Saensouk & Boonma var. nov

**Fig. 5:**
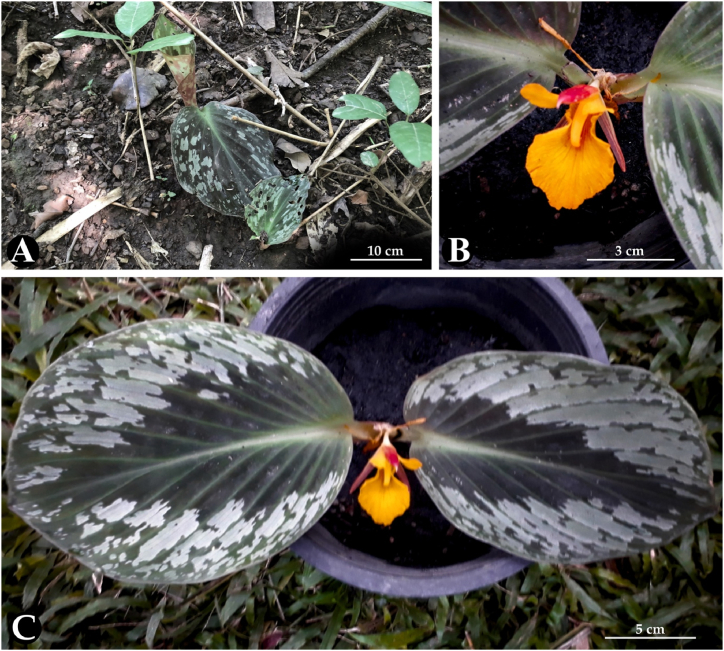
*Cornukaempferia aurantiiflora* var. *vespera* P. Saensouk, Saensouk & Boonma var. nov.; (A) habit in natural habitat; (B) top view of flower; (C) top view of habit (Photographs: A by Thawatphong Boonma, B–C by Surapon Saensouk).

**Fig. 6:**
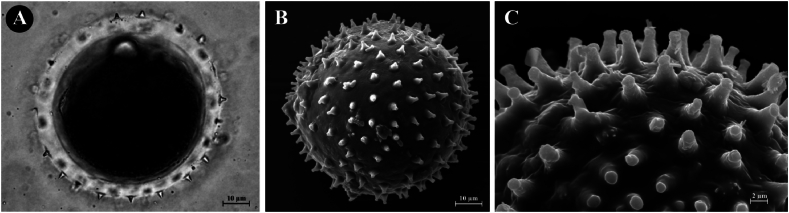
*Cornukaempferia aurantiiflora* var. *vespera* P. Saensouk, Saensouk & Boonma var. nov.; (A) Light Microscopy (LM) of pollen grain; (B) Scanning Electron microscopy (SEM) of pollen grain; (C) Scanning Electron microscopy (SEM) of exine sculpturing.

(Table 3, Fig. 5, Fig. 6(A–C))

**Table 3:** Morphological comparison of *C. aurantiiflora* var. *vespera* and var. *aurantiiflora*.

Characters	*C. aurantiiflora* var. *vespera*	*C. aurantiiflora* var. *aurantiiflora*
Anthesis time	In the evening, around 5:30 p.m.	In the morning, around 9:00 a.m.
Petiole	sessile or up to 3 cm long	5–11 cm long
Lamina	13–16 × 10–12 cm	20–25 × 14–15 cm
Calyx	10–11 mm long, with teeth 5–7 mm in length	c. 13 mm long, with teeth 7–9 mm in length
Floral tube	22–24 mm long	25–30 mm long
Corolla lobes	22–25 mm long	c. 40 mm long
Dorsal corolla lobe	24–25 mm long	c. 50 mm long
Lateral staminodes	23–25 × 9–10 mm	c. 30 × 13 mm
Labellum	30–32 × 22–25 mm	c. 45 × 35 mm

**Diagnosis:** The new variety, *Cornukaempferia aurantiiflora* var. *vespera*, resembles *C. aurantiiflora* Mood & K.Larsen var. *aurantiiflora* but differs in its evening anthesis time (around 5:30 p.m.), while the var. *aurantiiflora* blooms in the morning (approximately 9 a.m.). Additionally, this variety exhibits smaller or shorter characteristics compared to the var. *aurantiiflora*, including a rhizome diameter of 1.0–2.0 cm (vs. 1.5–2.0 cm for the var. *aurantiiflora*). Other distinguishing features include a sessile petiole or length of up to 3 cm long (vs. 5–11 cm long); lamina size of 13–16 × 10–12 cm (vs. 20–25 × 14–15 cm); calyx measuring about 10–11 mm long with teeth 5–7 mm in length (vs. a tubular calyx about 13 mm long with teeth 7–9 mm long); floral tube length of 22–24 mm (vs. 25–30 mm); corolla lobes measuring 22–25 mm long (vs. approximately 40 mm long); dorsal corolla lobe length of 24–25 mm (vs. approximately 50 mm), lateral staminodes measuring 23–25 × 9–10 mm (vs. approximately 30 × 13 mm), and a labellum size of 30–32 × 22–25 mm (vs. approximately 45 × 35 mm).

**Type**: Thailand, Uttaradit Province, Nam Pat District, c. 452 m a.s.l., *S.Saensouk 2022-25*, June 18, 2022 (holotype KKU!, isotype FOF!).

**Description:** Perennial rhizomatous herb. Rhizome 1.0–2.0 cm diam., yellowish white with a yellow core; tubers 1.0–1.2 × 5–7 cm, yellow with a light brown core, slightly aromatic. Leaves are usually 2 in number, *young leaf* is rolled and erect before unfurling, then lies slightly angled to the ground. Leaf-sheath 3–7 cm; ligule short, 2–4 mm emarginate; petiole sessile or short, length of up to 3 cm long, green with reddish brown tinge; lamina broadly ovate to suborbicular, 13–16 × 10–12 cm; upper surface green with riches silvery markings, glabrous with raised veins, lower surface dark purple with long, white hairs, margin undulating. Inflorescence few-flowered. Bracts lanceolate, acuminate, greenish with reddish apex, lower one c. 4 cm diminishing upwards; calyx tube 10–11 mm, teeth 5–7 mm; floral tube pale yellow, 22–24 mm; lobes dark reddish-orange; lateral lobes 22–25 × 6–8 mm, dorsal one 24–25 mm long, 9–10 mm at the base gradually tapering to the cucullate apex, margin incurved; lateral staminodes orange, oblanceolate with undulating margin, 23–25 × 9–10 mm; labellum orange with red lines at base, broadly deltoid, saccate, with undulating margin, 30–32 × 22–25 mm; stamen with very short filament, anther c. 15 mm long, opening longitudinally, connective produced into a long, narrow, forwards curved crest, 10–12 mm long. Epigynous glands 2, c. 5 mm long, yellow. Ovary ellipsoid, shortly hairy, c. 10 mm long. Fruits not seen.

**Vernacular name:** Proa Thong Baan Kaam.

**Distribution:** Endemic to Thailand.—Northern: Uttaradit Province, Nam Pat District (Fig. 1) **Etymology:** “*vespera*” is derived from the Latin word “*vesperum*”, meaning “evening”, which refers to its flower blooming in the evening.

**Ecology:** In the mixed deciduous forest on humus-rich soil, at an altitude of 450–560 m above sea level and is found growing alongside *Curcuma peramoena* Souvann. & Maknoi in the same area.

**Phenology:** Flowering in late May to July; anthesis time in the evening; normally flower starts to open around 5.30 p.m. and is closed and withered by 10 p.m.

**Palynology:** The pollen grains of *C. aurantiiflora* var. *vespera* are monad, inaperturate, spherical, with thick intine and thin exine with echinate sculpture, 71.83 ± 1.75 in diameter. The spine 3.18 ± 0.24 μm in length. The wall thickness 6.09 ± 0.27 μm (Fig. 6(A–C)).

**Utilization**: The species has been commercially sold and cultivated as an ornamental potted plant under the name *Cornukaempferia aurantiiflora* or “Proh Thong”.

**Conservation status**: In 2022, a newly identified variety was gathered, and the neighboring habitable regions are predominantly uninvestigated. Therefore, given the limited knowledge of its distribution range, it is plausible that this taxon could potentially inhabit surrounding, yet undiscovered, areas in other provinces. Consequently, according to the IUCN Red List Categories and Criteria [11], this variety has been classified as Data Deficient (DD). However, we recommend assuming that it is in danger of becoming extinct unless there is information to indicate otherwise.

**Fig. 7:**
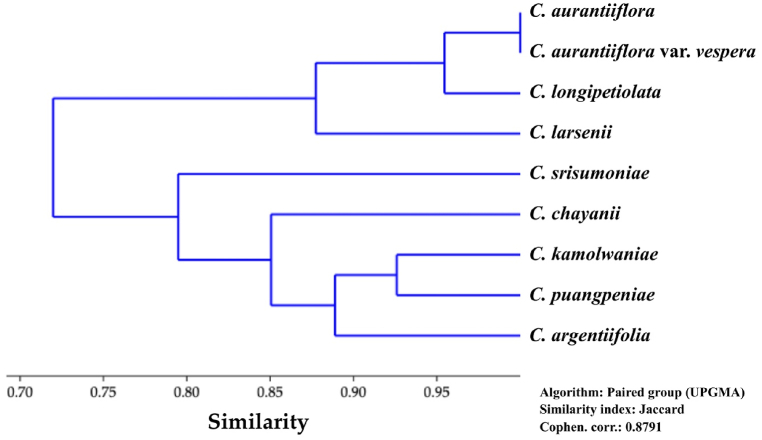
UPGMA cluster analysis dendrogram of similarity of *Cornukaempferia* taxa.

The dendrogram generated from UPGMA cluster analysis (Fig. 7) indicates that *Cornukaempferia aurantiiflora* var. *vespera* is most similar to the *C. aurantiiflora* var. *aurantiiflora*, and both taxa are forming a cluster alongside *C. longipetiolata* and *C. larsenii*. These taxa are distinguished by their leaves with silvery marks, glabrous staminodes, and glabrous labellum. While the newly described species, *C. puangpeniae*, exhibits morphological resemblances predominantly with *C. kamolwaniae*, closely followed by *C. argentifolia*, and forms a cluster alongside *C. chayanii* and *C. srisumoniae* within the same group, consistent with the group's shared characteristic of having hairy flowers, especially in the staminodes and labellum.

**Table:** Key to *Cornukaempferia* species and varieties

1a. Staminodes and labellum pubescent	2
1b. Staminodes and labellum glabrous	6
2a. Lamina adaxially plain green without marking	3
2b. Lamina adaxially silvery or with silvery marking	5
3a. Rhizome purple internally	*C. chayanii*
3b. Rhizome yellow internally	4
4a. Labellum apex rounded with incision; staminodes apex rounded	*C. kamolwaniae*
4b. Labellum apex acute without incision; staminodes apex acute	*C. puangpeniae*
5a. Filament <5 mm long; lamina silvery without marking; labellum apex emarginate	*C. argentifolia*
5b. Filament >5 mm; lamina green with silvery marking; labellum apex rounded	*C. srisumoniae*
6a. Lamina abaxially green	*C. larsenii*
6b. Lamina abaxially purplish	7
7a. Anther 2.8–3.3 cm long	*C. longipetiolata*
7b. Anther c. 1.5 cm long	8
8a. Anthesis in the morning	*C. aurantiiflora* var. *aurantiiflora*
8b. Anthesis in the evening	*C. aurantiiflora* var. *vespera*

#### 3.2.1 Preliminary altitude ranges of *Cornukaempferia* taxa

**Fig. 8:**
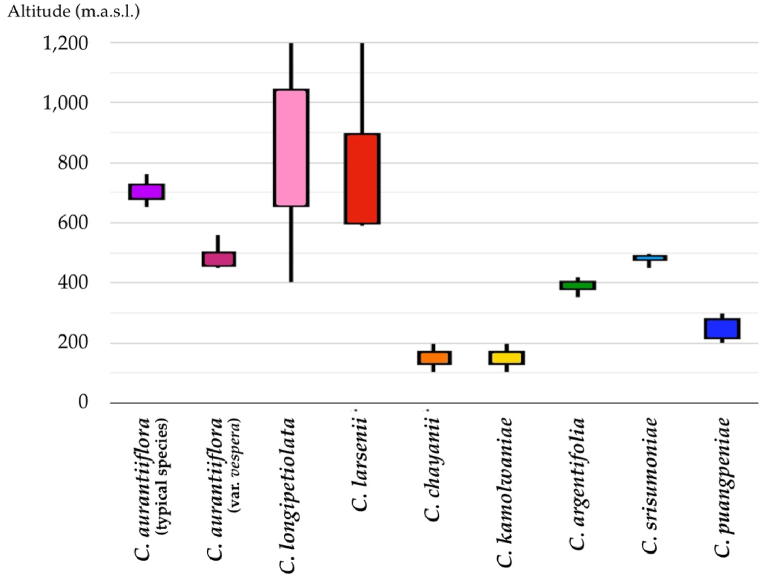
Box Plots of the preliminary altitude ranges of *Cornukaempferia* taxa.

The box plot visually depicts the preliminary altitude range of *Cornukaempferia* taxa, drawing from altitude data retrieved from the protologues of each taxon, our field expeditions, and data on herbarium specimen vouchers. Despite the constraints of limited data, this plot effectively showcases the diverse altitudes where each taxon was encountered, as illustrated in Fig. 8. However, this research may not provide a comprehensive range of altitudes from sea level for the distribution of each taxon. This limitation arises because many areas have limited access, and numerous suitable areas for this genus have not been surveyed or reported. Nevertheless, this box plot could help facilitate further study and exploration of these taxa. By combining this altitude range with distribution maps and altitude maps, it will be possible to roughly predict which areas may harbour additional populations of these taxa in the future.

Significant variations in altitudinal distributions are evident among the studied species. For example, *C. aurantiiflora* predominantly occurs within the altitude range of 650–765 m above sea level (m.a.s.l.), whereas *C. aurantiiflora* var. *vespera* inhabits lower altitudes, ranging from 450 to 560 m.a.s.l. In contrast, *C. longipetiolata* exhibits a notably wider altitudinal range, spanning from 400 to 1200 m.a.s.l. Conversely, species like *C. larsenii* demonstrate narrower altitudinal distributions, with populations primarily concentrated between 587 and 1200 m.a.s.l. Furthermore, lowland species such as *C. chayanii* and *C. kamolwaniae* are confined to altitudes ranging from 100 to 150 m.a.s.l., while transitional habitats at altitudes of 350–420 m.a.s.l. are favored by species like *C. argentifolia*. Moreover, *C. srisumoniae* is restricted to altitudes of 450–500 m.a.s.l., possibly influenced by microclimatic factors, whereas *C. puangpeniae* exhibit preferences for lower altitudes, ranging from 200 to 300 m.a.s.l. These distinct altitudinal patterns underscore the diverse ecological niches and adaptive strategies inherent within the species composition of the ecosystem under investigation.

In essence, the integration of altitudinal range data into ecological and conservation studies of *Cornukaempferia* species serves as a cornerstone for advancing scientific understanding, shaping conservation agendas, and guiding future research endeavors aimed at safeguarding these botanical treasures and the ecosystems they inhabit across diverse altitudinal ranges.

In exploring the potential of *Cornukaempferia* plants beyond their ornamental appeal, numerous opportunities emerge. One such avenue involves harnessing modern horticultural techniques, particularly tissue culture, to cultivate these plants. By adopting tissue culture methods, we can reduce the need to harvest them from their natural habitats, thus preserving biodiversity and ensuring sustainable sourcing. This approach not only safeguards wild populations but also allows for controlled cultivation, optimizing the quality and quantity of plant material for medicinal, commercial, and ornamental purposes. Investing in tissue culture infrastructure and expertise can pave the way for scalable production, furthering economic development while safeguarding environmental integrity. Thus, integrating tissue culture into horticultural practices presents a tangible solution to unlock the full potential of *Cornukaempferia* plants while promoting sustainable resource management and conservation.

#### 3.2.2 Discussion

The comparative analysis of morphological characteristics among various taxa within the *Cornukaempferia* genus reveals distinct patterns of differentiation and taxonomic relationships. Specifically, *Cornukaempferia puangpeniae* exhibits morphological similarities to *C. kamolwaniae* but displays significant divergences, particularly in dormancy traits and root anatomy. Notable distinctions include the presence of a tuberous root with two layers, differing from the multi-layered structure observed in *C. kamolwaniae*. Additionally, variations in leaf sheath length, lamina coloration, and floral morphology further delineate these species. These findings align with the observed clustering patterns in the UPGMA dendrogram, where *C. puangpeniae* clusters closely with *C. kamolwaniae* and shares similar hairy floral traits. This clustering reflects the taxonomic affinity inferred from morphological comparisons.

Conversely, newly described varieties such as *Cornukaempferia aurantiiflora* var. *vespera* exhibit morphological differences from *C. aurantiiflora* var. *aurantiiflora*, including temporal aspects of anthesis and various morphometric characteristics. These disparities underscore the importance of comprehensive morphological analysis in elucidating species diversity and taxonomic relationships within the *Cornukaempferia* genus. Integrating both qualitative and quantitative morphological data enhances our understanding of evolutionary dynamics and aids in precise species identification and classification within this taxonomic group.

The monad, inaperturate pollen grains with thin exine and thick intine, and the echinate sculpture observed in the two taxa, *Cornukaempferia puangpeniae* and *C. aurantiiflora* var. *vespera*, in this study are consistent with the findings reported by Zou et al. [12]. This alignment indicates a significant similarity in pollen morphology within the genus, corroborating the accuracy of prior taxonomic descriptions and contributing to a more detailed understanding of its botanical characteristics.

## 4 Conclusion

The unveiling of the new species *Cornukaempferia puangpeniae*, along with the new variety *C. aurantiiflora* var. *vespera*, alongside comprehensive morphological descriptions, pollen morphology, a revised key, and a dendrogram, significantly enriches our understanding of the *Cornukaempferia* genus. With the inclusion of these taxa, the genus now comprises eight species and two varieties, with a notable prevalence in Thailand, showcasing its significant endemism. Notably, *C. larsenii* stands out as native to both Thailand and Laos [6,10]. This research emphasizes the importance of ongoing botanical exploration and conservation efforts to preserve the rich biodiversity of the region and deepen our understanding of its flora.

## CRediT authorship contribution statement

**Piyaporn Saensouk:** Writing – review & editing, Writing – original draft, Visualization, Validation, Supervision, Software, Resources, Project administration, Methodology, Investigation, Formal analysis, Data curation, Conceptualization. **Surapon Saensouk:** Writing – review & editing, Writing – original draft, Visualization, Validation, Supervision, Software, Resources, Project administration, Methodology, Investigation, Funding acquisition, Formal analysis, Data curation, Conceptualization. **Thawatphong Boonma:** Writing – review & editing, Writing – original draft, Visualization, Validation, Supervision, Software, Resources, Methodology, Investigation, Formal analysis, Data curation, Conceptualization. **Chainarong Techa:** Resources, Investigation, Data curation. **Sarayut Rakarcha:** Resources, Methodology, Investigation. **Areerat Ragsasilp:** Resources, Methodology, Investigation. **Danh Duc Nguyen:** Methodology, Formal analysis, Data curation.

## Data availability statement

Data included in the article/supplementary material is referenced in the article.

## Ethics declarations

This study does not involve human participants, animals, or biological samples that would require ethical approval. All research activities complied with relevant institutional, national, and international guidelines for the study of plant species.

## Funding statement

This research project received financial support from 10.13039/501100020373Mahasarakham University.

## Declaration of competing interest

The authors declare the following financial interests/personal relationships which may be considered as potential competing interests:Surapon Saensouk reports financial support was provided by 10.13039/501100020373Mahasarakham University. Surapon Saensouk reports a relationship with 10.13039/501100007288Mahasarakham University that includes: employment, funding grants, and non-financial support. Surapon Saensouk has patent - pending to -. No If there are other authors, they declare that they have no known competing financial interests or personal relationships that could have appeared to influence the work reported in this paper.
